# Stereotactic body radiation therapy using CyberKnife for T1N0M0 lung cancer patients with severe pulmonary dysfunction

**DOI:** 10.1093/jrr/rraa075

**Published:** 2020-09-03

**Authors:** Takanori Abe, Yasuhiro Ryuno, Satoshi Saito, Tomomi Aoshika, Mitsunobu Igari, Ryuta Hirai, Yu Kumazaki, Kyoichi Kaira, Hiroshi Kagamu, Hironori Ishida, Shin-ei Noda, Shingo Kato

**Affiliations:** Departments of Radiation Oncology, International Medical Center, Saitama Medical University, Hidaka, Japan; Departments of Radiation Oncology, International Medical Center, Saitama Medical University, Hidaka, Japan; Departments of Radiation Oncology, International Medical Center, Saitama Medical University, Hidaka, Japan; Departments of Radiation Oncology, International Medical Center, Saitama Medical University, Hidaka, Japan; Departments of Radiation Oncology, International Medical Center, Saitama Medical University, Hidaka, Japan; Departments of Radiation Oncology, International Medical Center, Saitama Medical University, Hidaka, Japan; Departments of Radiation Oncology, International Medical Center, Saitama Medical University, Hidaka, Japan; Departments of Respiratory Medicine, International Medical Center, Saitama Medical University, Hidaka, Japan; Departments of Respiratory Medicine, International Medical Center, Saitama Medical University, Hidaka, Japan; Departments of General Thoracic Surgery, International Medical Center, Saitama Medical University, Hidaka, Japan; Departments of Radiation Oncology, International Medical Center, Saitama Medical University, Hidaka, Japan; Departments of Radiation Oncology, International Medical Center, Saitama Medical University, Hidaka, Japan

**Keywords:** CyberKnife, lung cancer, pulmonary dysfunction, stereotactic body radiotherapy

## Abstract

We retrospectively investigated the efficacy and safety of stereotactic body radiotherapy (SBRT) for T1N0M0 lung cancer using CyberKnife (CK) among 13 patients with severe pulmonary dysfunction which was defined as forced expiratory volume in 1 s (FEV_1.0_) of <1 L. The prescribed dose was 54 Gy in 3 fractions but adjusted for some patients if their tumors were in close proximity to the organs at risk (54 Gy/3 fractions: *n* = 11; 50 Gy/5 fractions: *n* = 1; 60 Gy/8 fractions: *n* = 1). During follow up (median follow-up: 27 months), we evaluated local control, overall survival and toxicity, using diagnostic imaging and laboratory tests. The patients’ median FEV_1.0_ was 0.84 L. Of the 13 patients, 3 were diagnosed as having lung cancer histologically and 10 diagnosed clinically. Their 2-year rates for overall survival and local control were 89 and 100%, respectively. So far, we have seen no adverse effects of grade 2 or higher. We concluded that CK-SBRT is effective and well tolerated for T1N0M0 lung cancer, even in patients with severe pulmonary dysfunction, but should be further evaluated with a larger cohort and longer follow-up periods.

## INTRODUCTION

Lung cancer is a leading cause of cancer death worldwide [1, 2]. For early-stage non-small-cell lung cancer, surgery is the established treatment [3]. However, some patients are not eligible for surgery due to comorbidities or advanced age. For such patients, stereotactic body radiotherapy (SBRT) reportedly offers good disease control with acceptable toxicity [4, 5]. CyberKnife® (CK; Accuray, Sunnyvale, CA, USA) is a specialized machine for stereotactic radiotherapy that allows highly conformal irradiation with tumor motion tracking [6], and is reportedly safe and effective for early-stage lung cancer [7–9]. SBRT with CK (CK-SBRT) can provide lower radiation doses to the lung than with conventional linac-based SBRT [10], which might be especially advantageous for lung cancer patients with pre-existing pulmonary dysfunction. In this study, we defined forced expiratory volume in 1 s (FEV_1.0_) < 1 L as severe pulmonary dysfunction (SPD). Absolute FEV_1.0_ is one commonly used predictive factor for radiation-induced lung toxicity [11]. Many clinical trials of SBRT for lung cancer are limited to patients with FEV_1.0_ > 0.7 L for participation [12, 13]. We think that patients with FEV_1.0_ 0.7–1 L is the lowest subset who may be candidates for SBRT for lung cancer. Here, we report outcomes of CK-SBRT for early-stage lung cancer patients with SPD.

## MATERIALS AND METHODS

### Patients

We retrospectively analyzed patients with T1N0M0 lung cancer and SPD (i.e., FEV_1.0_ < 1 L) who were treated by CK-SBRT at our institution. Most of these lung cancer patients were clinically diagnosed because their general condition was too poor to undergo biopsies and pathological confirmation. Clinical diagnoses were determined by multidisciplinary discussions, based on results of multimodal examinations, such as computed tomography (CT), ^18^F fluorodeoxyglucose-positron emission tomography (FDG-PET) and laboratory tests. Treatments such as surgery or radiation were also discussed by the cancer board. Cancers were staged using CT, FDG-PET and gadolinium-enhanced head magnetic resonance imaging, and classified by the Union for International Cancer Control (UICC) criteria (8th edition). This study was approved by our hospital’s institutional review board (No. 18–132).

### Treatment

The prescribed dose for the participants was 54 Gy in 3 fractions but was adjusted for some patients because of the proximity of the tumor to surrounding organs. If metallic marker insertion was feasible, we used fiducial markers to track tumor motion. If a patient’s condition was too poor to insert fiducial markers via bronchoscopy, the patient was irradiated under free breathing towards a target volume encompassing all of the tumor motion detected by 4D CT. Gross tumor volume (GTV) encompassing internal respiratory motion was created by the maximum intensity projection method with 4D CT. Planning target volume (PTV) was created with a 2-mm margin from GTV in every direction. The planning aim was to cover the GTV with 99% of the prescribed dose and to cover 90% of the PTV with 95% of the prescribed dose. The upper limit of the PTV maximum dose was 150% of the prescribed dose. The percentage of lung volume receiving >20 Gy (lung V_20_) should be <20%. Dose–volume parameters, including mean lung dose (MLD), lung V_20_, minimum dose in the most irradiated 99% of GTV (GTV-D_99_) and minimum dose in the most irradiated 90% of PTV (PTV-D_90_), both described as percentages of prescribed doses, were evaluated and recorded prior to treatment.

### Evaluation

Patients were followed up every 3 months for the first year and every 6 months thereafter. Chest X-rays, CTs and laboratory tests were taken periodically. Acute toxicity was defined as adverse events (AEs) that occurred within 90 days after treatment; late toxicity was defined as AEs that occurred thereafter. All AEs were classified by the National Cancer Institute Common Toxicity Criteria for Adverse Events version 4.0. Overall survival (OS) was defined as the interval from start of treatment to death or last follow-up date. Local control was defined as no local recurrence in the irradiated field.

### Statistical analysis

Cumulative OS rate and local control rate were calculated using the Kaplan–Meier method. Mean parameters in two groups were compared with Student’s *t* test. These statistical analyses were performed using IBM SPSS Statistics for Windows, Version 25.0 (SPSS Inc., Armonk, NY, USA).

## RESULTS

### Patient and treatment characteristics

We analyzed 13 patients (6 men and 7 women) with T1N0M0 lung cancer who had SPD (FEV_1.0_ < 1 L) and were treated with CK-SBRT at our hospital between July 2015 and May 2019. Their median age was 75 years (range: 71–85 years). Their median follow-up was 27 months (range: 5–55 months) and median the FEV_1.0_ was 0.84 L (range: 0.49–0.9 L). Diffusing capacity for carbon monoxide (DLCO) was measured in 9 patients. Among 9 patients, median %DLCO, which was calculated by dividing absolute DLCO by predicted DLCO, was 22.5% (range: 14.5–107.4%). Lung vital capacity (VC) was measured in all patients and %VC, which was calculated by dividing absolute VC by predicted VC, was 78.7% (range: 64.5–90.0%). Regarding the cause of pulmonary dysfunction, 11 patients had a history of smoking, 1 patient had a history of traumatic hemopneumothorax and 1 patient was a super elderly (87 years old), small, female patient. None of the patients in this study had a history of chronic infectious lung disease such as tuberculosis and atypical mycobacteriosis nor interstitial lung disease. Four patients had already received home oxygen therapy (HOT) before SBRT. Histological diagnosis of lung cancer was confirmed in 3 patients (adenocarcinoma, squamous cell carcinoma and non-small cell lung cancer: *n* = 1 for each) and it was clinical in 10 patients using multimodal examinations. According to the UICC staging system, 6 patients had T1bN0M0 and 7 had T1cN0M0. Their median tumor diameter was 21 mm (range: 14–21 mm). The prescribed SBRT doses were 54 Gy in 3 fractions (*n* = 11), 50 Gy in 5 fractions (*n* = 1) and 60 Gy in 8 fractions (*n* = 1). Mean lung values were lung V_20_: 3.4%, GTV-D_99_: 98.2%, PTV-D_90_: 96.9% and maximum PTV dose: 137.7% of the prescribed dose. Tumor motion tracking with fiducial marker was used in 3 patients. These characteristics are summarized in Table 1.

**Table 1:** Patient and treatment characteristics (*N* = 13)

Characteristic		*n* (%)
Age, years^a^		75 (71–85)
FEV_1.0_, L^a^		0.84 (0.49–0.9)
FEV_1.0_, % as predicted^a^		39.7 (27.5–72.1)
Blinkman Index^a^		800 (0-1680)
Home oxygen therapy	Yes	4 (31)
	No	9 (69)
Sex	Male	6 (46)
	Female	7 (54)
Histopathological type	Adenocarcinoma	1 (8)
	Squamous cell carcinoma	1 (8)
	Non-small cell lung cancer	1 (8)
	Not identified	10 (76)
T classification	T1b	6 (46)
	T1c	7 (54)
Diameter of tumor, mm		21 (14–28)
Radiation dose	50 Gy in 5 fractions	1 (8)
	54 Gy in 3 fractions	11 (84)
	60 Gy in 8 fractions	1 (8)
Tumor motion tracking	Yes	3 (23)
	No	10 (77)
Lung V20, %^b^		3.4 (±2.5)
Mean lung dose, Gy^b^		3.2 (±1.4)
GTV D99, %^b^^,^^c^		98.2 (±4.0)
PTV D90, %^b^^,^^c^		96.9 (±4.3)
Maximum dose in PTV, %^b^^,^^c^		137.7 (±11.6)

a Shown as median (range).

b Shown as mean (± Standard deviation).

c described as percentage for prescribed dose.

FEV_1.0_ = forced expiratory volume in one second, Lung V20 = normal lung volumes that received more than 20 Gy, GTV D99 = minimum dose in most irradiated 99% of gross tumor volume described as percentage for prescribed dose, PTV D90 = minimum dose in most irradiated 90% of planning target volume described as percentage for prescribed dose.

### Clinical results

**Fig. 1.**
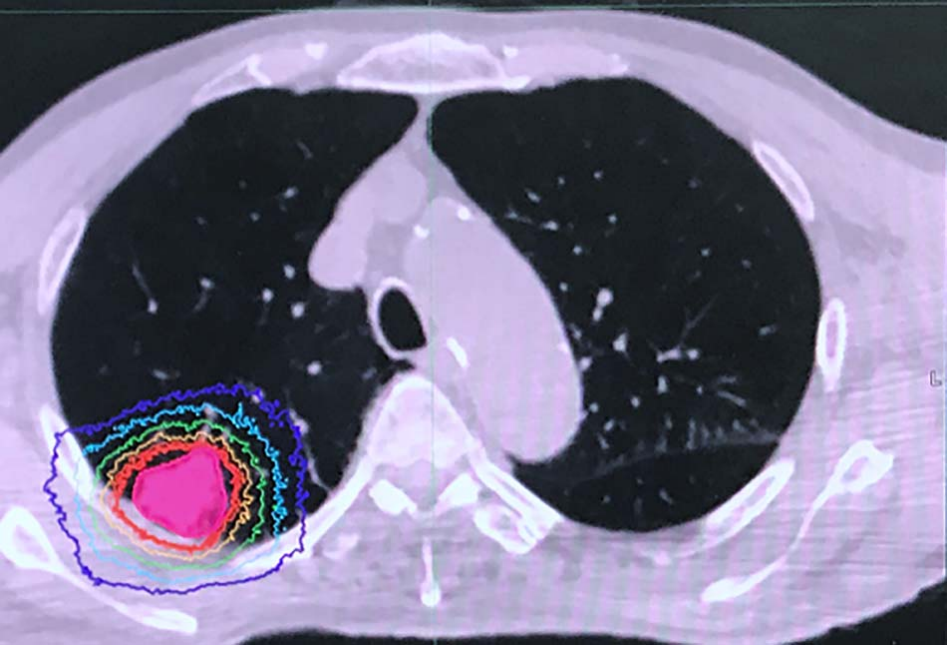
Dose distribution of a representative patient. Prescribed dose was 54 Gy in 3 fractions. The thick red line shows 95% of the prescribed dose and the thick purple line shows the 20 Gy isodose line. Gross tumor volume is shown with pink mesh.

**Fig. 2.**
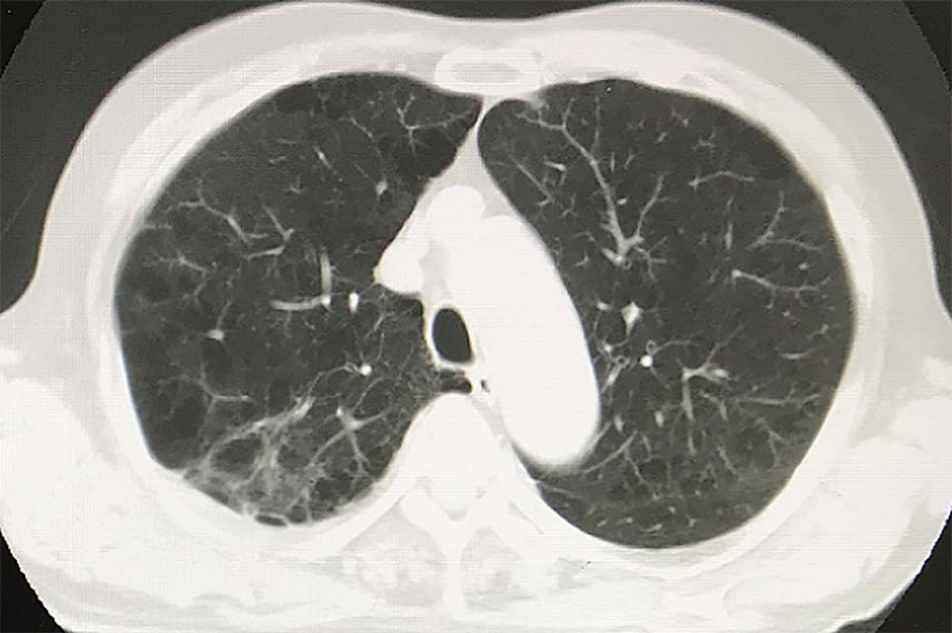
CT image of same patient as in Fig. 1 taken 27 months after treatment showed no evidence of disease.

**Fig. 3.**
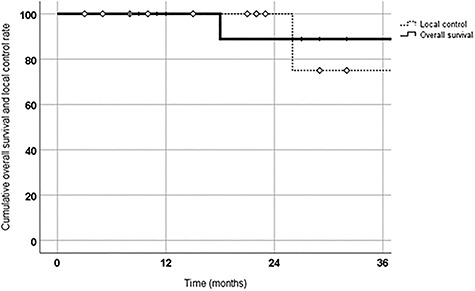
Cumulative OS rate and local control rate are shown. The 2-year OS rate and local control rate were 89 and 100%, respectively.

A representative case is shown in Figs 1 and 2. Median survival time was 27 months. At last follow up, 2 patients had died—one of aspiration pneumonia at 38 months after treatment and the other of newly developed small cell lung cancer at 18 months after treatment. One patient developed local recurrence at 26 months after treatment. Cumulative 2-year OS and local control rates were 89 and 100%, respectively (Fig. 3). Pre- and post-treatment saturation of percutaneous oxygen (SpO_2_) data were available for 10 patients. Mean pre-treatment SpO_2_ was 96.1% and mean post-treatment SpO_2_ was 95.9% (*P* = 0.296). The median interval between pre- and post-treatment SpO_2_ measurement was 24 months. All the patients were treated as outpatients safely, with no acute AEs. As a late AE, grade 1 radiation pneumonitis (RP) was observed in 7 patients but no grade 2 or higher RP was observed. The median duration to develop RP was 5 months. No patient started HOT after SBRT.

## DISCUSSION

Although the median FEV_1.0_ among our patients was 0.84 L, their respective cumulative 2-year OS and local control rates were 89 and 100% and we saw no AEs of grade 2 or higher.

The natural course of patients with SPD should be considered in determining optimal treatment. Górecka *et al*. reported the 2-year OS rate to be 77% in their randomized control trial to clarify the effects of long-term oxygen therapy on patients with chronic obstructive pulmonary disease (COPD) with moderate hypoxemia [14]. In their study, the median FEV_1.0_ was 0.83 L, which was similar to our study. The 2-year OS rate in our study was 89%, which indicates that, at least, our treatment did not worsen patient prognosis. In this study, mean pre-treatment SpO_2_ was 96.1% and mean post-treatment SpO_2_ was 95.9% (*P* = 0.296), with a median interval of 24 months between pre- and post-treatment measurement. This result also indicates that CK-SBRT has low invasiveness for lung cancer with SPD. Although optimal treatment is affected by the patient’s general condition, tumor aggressiveness and the patient’s cooperation, CK-SBRT is an attractive option for lung cancer patients with SPD.

In this study, 1-year and 2-year OS rates were 100 and 89%, respectively. Hara *et al*. reported that respective 1-year and 2-year OS rates were 87 and 70% after 40–60 Gy in 5 fractions of linac-based SBRT for lung cancer with COPD [15]. Temming *et al.* reported that the 2-year OS rate was 77% after CK-SBRT for patients with early-stage lung cancer and pulmonary dysfunction [7]. Results of SBRT for lung cancer patients with pulmonary dysfunction are summarized in Table 2 [16, 17]. We believe our result are comparable to these studies, although our patients had SPD.

**Table 2:** Treatment efficacy of stereotactic body radiotherapy for lung cancer patients with pulmonary dysfunction in the literature

Authors	Number of patients	Pre-treatment pulmonary function	Total dose/fractions	Treatment machine	Treatment outcome
Hara et al. [12]	24	Median FEV_1.0_ = 1.12 L	40-60 Gy/5 fractions	Linac	3y-OS 49%, 3y-LC 93%
Palma et al. [13]	176	Median FEV_1.0_ = 0.94 L	54-60 Gy/3-8 fractions	Linac	3y-OS 47%, 3y-LC 89%
Temming et al. [7]	106	NA	51-60 Gy/3-8 fractions	CyberKnife	2y-OS 77%, 2y-LC 88%
Present study	13	Median FEV_1.0_ = 0.84 L	50-60 Gy/3-8 fractions	CyberKnife	2y-OS 89%, 2y-LC 100%

FEV_1.0_ = forced expiratory volume in one second, OS = overall survival, LC = local control, NA = not applicable.

We saw no AEs of grade 2 or higher in this study. Treatment-related lung AEs and deterioration of daily living activities were the most concerning problems after SBRT for lung cancer patients with SPD. Reported incidences of grade ≥ 2 RP were 3–28% after SBRT for lung cancer patients with pulmonary dysfunction [7, 18]. In our study, lung V_20_ was 3.5% and MLD was 3.2 Gy, which were adequately low. The AE profile and treatment details in the literature are summarized in Table 3. We believe 54 Gy in 3 fractions of CK-SBRT is safe even for patients with SPD.

**Table 3:** Toxicity profile in the literature

Authors	Number of patients	Pre-treatment pulmonary function	Treatment machine	Dose-volume parameters of the lung	Lung toxicity
Hara et al. [12]	24	Median FEV_1.0_ = 1.12 L	Linac	Median MLD =3.3 Gy	Grade ≥ 2 = 17%
Baumann et al. [14]	60	Mean FEV_1.0_ (%) = 49	Linac	Median V20 = 8-12%,	Grade 1,2 = 17.5%
				Median MLD = 6-8 Gy	
Temming et al [7]	106	NA	CyberKnife	NA	Grade 2 = 3%
Present study	13	Median FEV_1.0_ = 0.84 L	CyberKnife	Mean V20 = 3.4 %	Grade ≥ 2 = 0%
		Median FEV_1.0_ (%) = 40		Mean MLD = 3.2 Gy	

FEV_1.0_ = forced expiratory volume in one second, MLD = mean lung dose, V20 = normal lung volumes that received more than 20 Gy, NA = not applicable.

Only 1 of our patients developed local recurrence. This patient was treated with a total SBRT dose of 60 Gy in 8 fractions because of the tumor’s proximity to the heart. Although few data are available on the relationship between SBRT dose to the heart and cardiac toxicity for early-stage lung cancer, Stam *et al*. reported that doses to the heart were associated with non-cancer death [19]. We adopted a conservative policy regarding dose to the heart, in view of the lack of solid evidence. Investigations of optimal dose fractionation schedules for good local control of thoracic tumors while avoiding cardiac toxicity are needed. Other patients in our study who were treated with 54 Gy in 3 fractions of SBRT have not shown local recurrence to date. Stephans *et al*. reported that a significantly lower rate of local failure was observed after 54–60 Gy in 3 SBRT fractions than with 48–50 Gy/4–5 fractions, or with 50–60 Gy/8–10 fractions [20]. We consider that 54 Gy in 3 fractions produces good local control, even for patients with SPD. Additionally, our planning aim and dose constraint, such as to cover 99% of GTV with the prescribed dose and to allow high maximum dose to the PTV, may also improve local control, although longer follow-up with a larger study cohort is necessary to clarify the dose effect on local control and OS.

This study had some limitations. First, it was a retrospective analysis with a small number of patients. Second, most of the patients were not histologically diagnosed, which may have ultimately affected treatment outcomes such as local control and OS.

In conclusion, CK-SBRT is effective and well tolerated for stage T1N0M0 lung cancer patients even with SPD. However, our findings should be verified with a larger cohort and longer follow-up.

## CONFLICT OF INTEREST

None declared.

## FUNDING

The authors have no funding source to be declared. 
